# Examining the dualistic model of passion in addiction recovery

**DOI:** 10.3389/fpubh.2025.1519430

**Published:** 2025-02-26

**Authors:** Lauren E. Lewis, Devin J. Mills, Brandon G. Bergman, Thomas G. Kimball, William Gerber

**Affiliations:** ^1^School of Medicine, University of Connecticut, Farmington, CT, United States; ^2^Community, Family, and Addiction Sciences, Texas Tech University, Lubbock, TX, United States; ^3^Recovery Research Institute, Massachusetts General Hospital & Harvard Medical School, Boston, MA, United States

**Keywords:** addiction recovery, substance use, dualistic model of passion (DMP), passion, recovery capital

## Abstract

**Introduction:**

Addiction recovery can be conceptualized as multidimensional changes to health and wellness including changes in substance use, physical and mental health, and social relationships. These outcomes are often measured through recovery capital which recognizes the various resources, both internal and external, that one may use to enhance their recovery. Internal and external resources can also be accumulated by engaging in an activity an individual is passionate about, explained by the dualistic model of passion (DMP) as enhancing mental and spiritual well-being, health, and personal growth, thereby fostering positive emotions, community involvement, deeper relationships, and heightened performance across various life domains. Evidence indicates that both RC and the DMP contribute to improved health outcomes including life satisfaction and well-being; however, the DMP has not yet been applied to addiction recovery science. The current study aimed to contribute to the growing body of research on addiction recovery by exploring the way passion may influence recovery outcomes. Further, the study investigated how the differences in passion type [e.g., harmonious (HP) and obsessive (OP)] impacted RC.

**Methods:**

Participants for the study (*N* = 346; *M*_age_ = 42.1; 53.2% Male) included individuals who self-reported being in recovery from alcohol and/or drugs and completed an online survey through Amazon’s Mechanical Turk platform.

**Results:**

A significant bivariate correlation was found between HP and RC (*r* = 0.42, *p* < 0.001), and mean comparisons showed significant differences for individuals endorsing HP as they scored higher on a measure of RC (*M* = 4.69) than did their peers endorsing OP (*M* = 4.26). Finally, a regression analysis found that HP predicted RC (*B* = 0.19, *p* < 0.001), even when time in recovery and mental health variables such as depression and anxiety were included in the model.

**Discussion:**

This study offers novel evidence for an association between the DMP and recovery outcomes warranting future research.

## Introduction

The National Survey on Drug Use and Health conducted yearly by the Substance Abuse and Mental Health Services Administration (1) found that of the 30.5 million adults who ever believed they had a problem with their use of drugs or alcohol, 73.1% considered themselves to be in recovery or recovered from their problem. Historically, recovery research focused on acute outcomes by measuring days abstinent or drinks per day within 1 year after treatment (2); however, researchers, clinicians, and people with lived experience have recently advocated to expand the way recovery outcomes are defined and measured. To include the wide breadth of transformations undergone by an individual including changes to their substance use as well as their overall health and well-being, current conceptualizations recognize that recovery is a unique and individualized process (3–5). The current paper defines recovery as “a process of change through which individuals improve their health and wellness, live a self-directed life, and strive to reach their full potential” (6).

This new conceptualization of addiction recovery has required a fundamental shift in theory and research related to the ways people achieve wellness and gain control over their health, therefore recovery models and assessments must acknowledge the various pathways and outcomes individuals in recovery may experience. Recovery capital (RC) (7, 8) is a common and comprehensive approach to understanding and measuring recovery from this modern perspective. RC states that the initiation and maintenance of addiction recovery can be better understood by identifying an individual’s resources, defined as “capital” which they accumulate over time. The most current RC model, which will be used in the current paper, identifies the sum of resources found within four domains: social, physical, human, and cultural capital (8). These resources include positive outcomes gained in recovery extending beyond abstinence such as feeling supported in social relationships, being satisfied with life, feeling connected to a community, and feeling an enthusiasm for life (9). These same outcomes have been found when an individual engages in an important, self-defining activity, known as a passion (10).

Passion, as defined by the dualistic model of passion (DMP) is a “strong inclination toward a specific object, activity, concept, or person that one loves (or at least strongly likes), highly values, invests time and energy in on a regular basis, and that is part of one’s identity” [(11), p. 33]. When an individual engages in their passion they often experience heightened affect, connection to a social environment, and a strong motivation. Within this model two types of passion exist, harmonious (HP) and obsessive (OP). Individuals who engage in their passion harmoniously are able to participate with openness, flexibility, and mindfulness where they remain in control of the activity without it overpowering their time (11). Conversely, OP is driven by an uncontrollable urge, in which their passion is in conflict with other aspects of life and often experience a sense of insecurity, failure, and shame (11).

Research on the DMP has long examined the differing results of HP and OP consistently finding that HP leads to adaptive outcomes, whereas OP is associated with maladaptive consequences (10, 12, 13). A recent meta-analysis conducted by Curran et al. (14) highlights these findings through the review of research on the DMP and intrapersonal outcomes (e.g., well-being, cognitive experiences, and motivation). The review found consistent evidence that HP is related to increased well-being, life satisfaction, and vitality, whereas OP is associated with ill-being through negative affect and burnout. These findings have also been underscored in research on passion for using substances (e.g., passion for alcohol use) which reports that OP is related to higher consumption, consequences, and craving, while HP serves as a protective factor (15–18). While research on the DMP and substance use has started to develop, to date, research has not been published on the effect of passion on addiction recovery. We argue that passion likely plays an important role in the recovery journey, but that its effect is dependent upon the type of passion, offering a potentially useful construct to teach us novel ways to impact recovery.

The current study aims to explore the relationship between RC and the DMP using an online sample of people in recovery from alcohol and/or substance use. Given the consistent evidence that HP contributes to higher well-being, and the contemporary approach to measuring well-being as a key component of the overall changes experienced during recovery, it can be assumed that HP will positively contribute to recovery outcomes. First, we will test the differences between scores of RC and mental health based on individuals endorsing HP versus OP. We hypothesize that those endorsing HP will have higher averages of RC and lower scores on indices of mental health. Second, we will test the differences between those who did not endorse a passion at all (NP), and the HP, OP groups. We hypothesize that the NP and OP group will both report lower RC and higher mental health indices. Third, we will use a regression analysis to test the effects of passion on RC, and we hypothesize that HP will predict higher RC, while OP will contribute to lower levels of RC. Finally, we hypothesize that passion will continue to predict RC even when other related variables are included in the model including time in recovery, mental health, and life satisfaction.

## Methods

### Procedures

US adults with a minimum 90% approval rating on MTURK were screened for being 18 years or older, a U.S. resident, and in recovery from alcohol and/or drugs or having resolved a problem with alcohol and/or drugs. Participants who answered yes to all three questions (*N* = 514) were invited to complete a survey on substance use and recovery history, passion, depression, anxiety, life satisfaction and other related variables. Participants were compensated through MTURK for their time completing both surveys. To ensure integrity of the data, the sample was reduced based on the following criteria: (1) Incorrectly responding to two out of three attention check questions, (2) Inconsistent response to the two recovery status questions between the screening and final survey, and (3) completing the survey in five minutes or less (38). The total number of participants who completed the survey (*n* = 393) were reduced to a final sample (*n* = 346) based on these measures.

### Participants

The participants (*N* = 346) mean age was 42.1 years (range: 20–75), who were assigned male at birth (53.2%) and Caucasian/white (82.1%) as seen in Table 1. The sample represented individuals in recovery with 80.3% self-identifying as currently being in recovery, and 95.1% reporting that they used to have a problem with drugs and/or alcohol but no longer do. Just over half of the sample (54.6%) reported being in recovery from alcohol only, while 20.5% reported being in recovery from drugs, and 24.9% in recovery from both. Participants who reported having a passion (*n* = 245) represented 71% of the sample in line with previous reports of the general population (10, 12).

**Table 1:** Sample characteristics (*N* = 346).

	*n*	*%*
Age	*M* = 42.1	*SD* = 12.8
Sex		
Male	184	53.2
Female	162	46.8
Race		
Caucasian/white	284	82.1
Hispanic/Latino	26	7.5
African American/black	39	11.3
Asian American/Asian	17	4.9
American Indian/Native Alaskan	9	2.6
Native Hawaiian/Pacific Islander	2	0.6
Income		
Less than $50,000	190	55
$50,000 to $99,999	122	35.3
$100,000 or more	34	9.8
Current recovery		
Alcohol only	189	54.6
Drugs only	71	20.5
Both alcohol and drugs	86	24.9
Time in recovery^a^		
< 1 year	97	28
1–5 years	130	37.6
5–10 years	46	13.3
> 10 years	73	21.1
Natural recovery		
Yes	78	22.5
No	268	77.5
Mutual-help attendance		
Yes	115	33.2
No	231	66.8
Recurrence of use in last 6 months		
Yes	131	37.9
No	215	62.1

a For individuals in recovery from both alcohol and drugs, shorter duration of recovery is reported.

### Measures

#### Recovery history

Participants current recovery status was measured with two questions in both the screener and full survey including if they “used to have a problem with alcohol and/or drugs but no longer do,” and “consider themselves to currently be in the process of recovery” (19). Participants who responded yes to at least one of the questions were included in the final sample. Next, they were asked to identify whether their recovery was related to alcohol and/or drugs with the option to choose both. To assess time in recovery the participants were given seven options ranging from *Less than 6 months* to *More than 15 years*. For participants who reported varying periods of time in recovery from alcohol and drugs simultaneously, the lowest time was used in final analysis (e.g., Individual is in recovery from alcohol for 5–10 years, but drugs for 6–12 months, the final assessment would reflect recovery time as 6–12 months). Other items assessing recovery history included current substance use, the types of programs or services used to aid in their recovery journey, and any recurrence of use within the last 6 months.

#### Passion

Passion was measured using the 17-item Passion Scale (10). Participants were first given a definition of recovery and asked if they had an activity they would define as a passion. Those who answered yes (*n* = 245) were asked to identify their passion with an open-ended response. Next, they rated six items assessing HP (e.g., “*my activity is well integrated in my life*;” *α* = 0.80) and OP (e.g., “*I have difficulties controlling my urge to do my activity;*” α = 0.74) along with five items measuring passion criteria (e.g., “*This activity is important for me;*” α = 0.74). The questions are rated on a 7-point Likert scale ranging from *Do not agree at all* (1) to *Very strongly agree* (7). The average score for each subscale was used in the final analysis. Additionally, participants were classified into either HP or OP based on their higher sub-scale z-score for statistical analyses of group comparison (20). An additional question was asked in which participants rated how much they believed engaging in their passion supported their recovery rated from *Strongly disagree* (1) to S*trongly agree* (7), to which 69.6% agreed at some level.

#### Recovery capital

Recovery capital was measured using the Assessment of Recovery Capital (ARC) (21). The assessment contains 50 items with a dichotomized response (e.g., yes or no), but the current study utilized a 6-point Likert scale similar to the Brief Assessment of Recovery Capital (BARC-10) (9) ranging from *Strongly disagree* (1) to *Strongly agree* (6). Items include a wide variety of statements such as “I am currently completely sober,” and “I feel physically well enough to work.” Each participant’s average score was used in the final analysis (*α* = 0.96).

#### Mental health

Mental health was assessed using the Patient Health Questionnaire-8 (PHQ-8) (22) and the Generalized Anxiety Disorder Assessment (GAD-7) (23). Both measures have been found to be consistent and reliable measures of depression and anxiety. The PHQ-8 uses eight items based on the participant’s experience of depression criteria (e.g., “*Feeling down, depressed, irritable or hopeless”*) within the last 2 weeks. It is rated on a 4-point Likert scale and the total score is used in analysis (*α* = 0.89). The GAD-7 also asks participants to rate how often seven anxiety items (e.g., “*Worrying too much about different things”*) have affected them in the last 2 weeks. The assessment uses a 4-point Likert scale, and the total score was used in the current analysis (α = 0.93).

#### Satisfaction with life

A 5-item assessment of life satisfaction was utilized (SWLS) (24) where participants are asked to rate the statements on a scale from (1) *Strongly disagree*, to (7) *Strongly agree*. The items (e.g., “*In most ways my life is close to my ideal*”) assess overall satisfaction, and the measure has consistently been found to be reliable and valid (α = 0.91).

### Analysis

**Figure 1:**
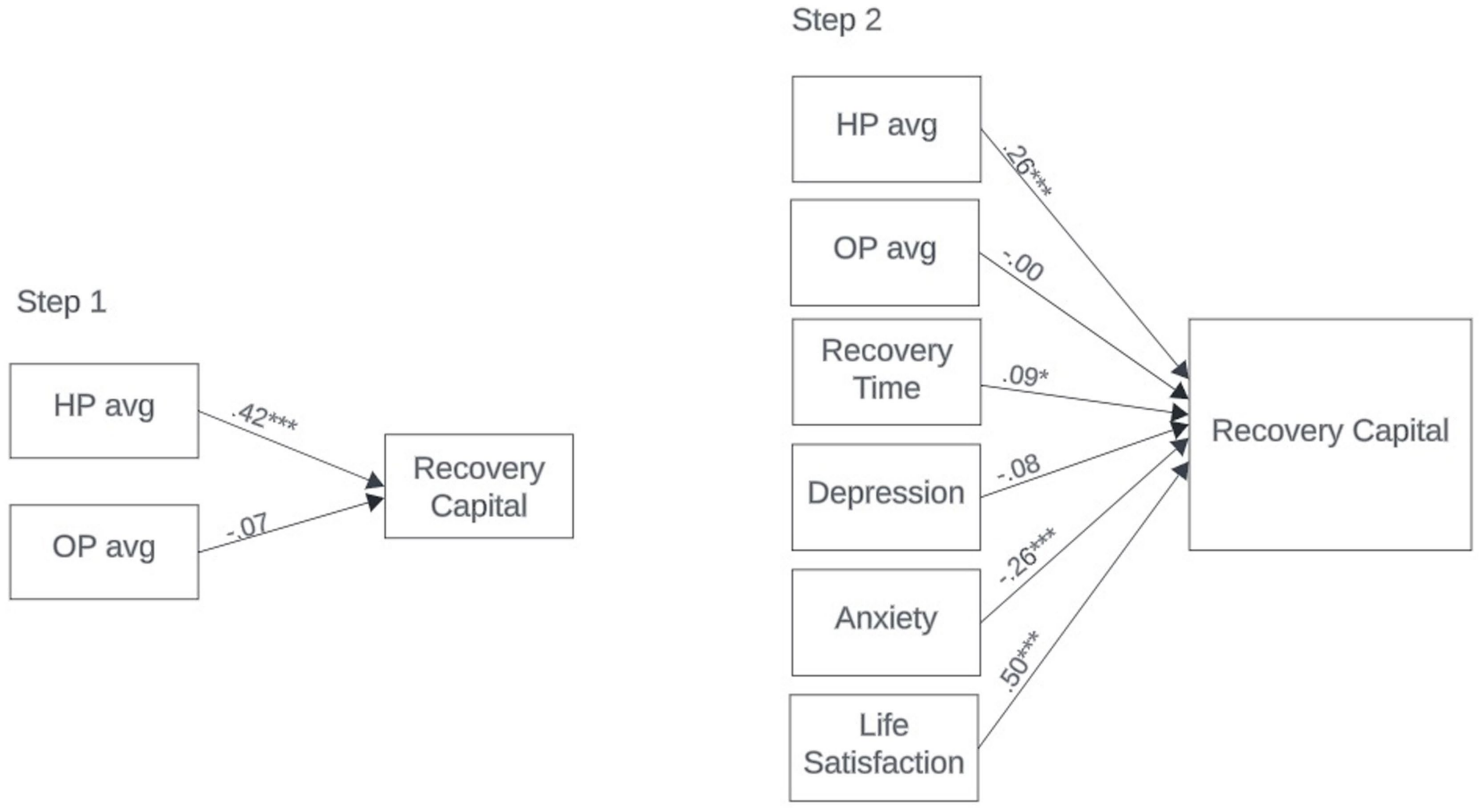
Standardized model results for hierarchical linear regression.

Initial data preparation was completed using SPSS (Version 29.0). Data analysis including basic descriptive statistics, bivariate correlations, *t*-tests, and analysis of variance (ANOVA) were completed with the final sample (*n* = 346). After testing for equal variance revealed that equal variance could not be assumed, the ANOVA analysis included post-hoc tests (e.g., Games-Howell, and Tamhane) to confirm any significant relationships (25). Finally, a two-step hierarchical regression analysis was conducted in SPSS only accounting for the sub-sample of individuals who reported having a passion (*n* = 245) employing listwise deletion of participants with missing data (*n* = 236), to test the effect of passion on recovery capital. Missing data accounted for less than 5% of the total sample, introducing minimal bias with negligible effects on the results (26). The first step included the average scores for HP, OP, and RC, and the second step additionally included time in recovery and other measures of well-being including satisfaction with life, depression, and anxiety to further assess the associations (See Figure 1).

## Results

### Associations between passion, recovery, and mental health

Preliminary analysis was conducted to estimate associations between the study variables (see Table 2). Bivariate correlations were calculated showing HP was significantly positively correlated with RC, while OP was negatively correlated and non-significant. To test our first hypothesis, *t*-tests were conducted to compare the differences in recovery outcomes for individuals endorsing HP compared to OP and revealed significant differences in RC scores *t* (242) = 4.80, *p* < 0.001 (See Table 3). Both bivariate correlations and the *t-tests* confirmed that HP is related to lower depression *t* (242) = 5.87, *p* < 0.001 and anxiety *t* (242) = 4.37, *p* < 0.001. As hypothesized, individuals in the HP group had significantly higher RC scores than those in the OP group. The HP group also experienced less symptoms of both depression and anxiety indicating that in line with previous research, those engaging in a passion harmoniously may also possess more recovery resources and experience improved mental health than their peers.

**Table 2:** Descriptive statistics and bivariate, and partial correlations between study variables.

Variable	*M*	*SD*	1	2	3	4	5	6
1. Recovery capital	4.32	0.77	___	0.38**	0.06	___	___	___
2. Harmonious passion	5.68	0.97	0.42**	___	0.17*	___	___	___
3. Obsessive passion	3.50	1.69	−0.02	0.14*	___	___	___	___
4. Time in recovery	3.78	1.88	0.21**	0.07	−0.06	___	___	___
5. Depression	7.92	5.71	−0.52**	−0.15*	0.34**	−0.20**	___	___
6. Anxiety	7.13	5.90	−0.55**	−0.11	0.28**	−0.13*	0.77**	
7. Life satisfaction	19.58	7.97	0.70**	0.20**	0.09	0.09	−0.36**	−0.35**

*p < 0.05. **p < 0.01. Upper diagonal represents partial correlations after controlling for variables 4–7. Time in recovery based on ordinal variable indicating average time in recovery is between 2 to 5 years.

**Table 3:** Results of *t*-tests when comparing study variables across passion type.

Variable	HP^a^	OP^a^			
	*M (SD)*	*M (SD)*	*t*	*p*	*Cohen’s d*
Recovery capital	4.69 (0.67)	4.26 (0.71)	4.80	<0.001	0.61
Time in recovery	4.15 (1.92)	3.73 (1.75)	1.79	0.07	0.23
Depression	5.42 (4.80)	9.42 (5.81)	5.87	<0.001	0.75
Anxiety	4.99 (5.59)	8.17 (5.59)	4.37	<0.001	0.57
Life satisfaction	22.04 (7.63)	20.27 (7.58)	1.82	0.07	0.23

a Based on Z-score categorizations.

To test our second research questions, further mean comparisons explored the differences between HP (*n* = 120), OP (*n* = 124), and individuals who did not indicate a passion (NP; *n* = 99) using ANOVA procedures (see Table 4). Results show that individuals endorsing HP have significantly higher RC than those without a passion and those endorsing OP *F* (2, 343) = 31.01, *p* < 0.001, in line with our hypothesis. Individuals endorsing OP did have significantly more RC than those who did not have a passion at all. These findings point to the fact that engaging in a passion at all may contribute positively to recovery resources, while engaging in the passion harmoniously offers the greatest benefit. Additionally, HP was significantly lower than OP and NP for both depression *F* (2, 343) = 19.53, *p* < 0.001 and anxiety *F* (2, 331) = 12.79, *p* < 0.001.Lastly, satisfaction with life scores *F* (2, 341) = 19.79, *p* < 0.001 were highest for those endorsing HP and significantly different from NP, but not OP. These findings offer preliminary evidence for the differences between HP and OP while also adding evidence for the similarities between OP and non-passionate individuals.

**Table 4:** Means, standard deviations, and one-way ANOVA statistics for study variables.

	NP (*n* = 101)	HP (*n* = 120)	OP (*n* = 125)			
	*M (SD)*	*M (SD)*	*M (SD)*	*F*(2,343)	*η^2^*	Significant differences (*post hoc*)
Recovery capital	3.93 (0.75)	4.69 (0.67)	4.26 (0.71)	31.01***	0.15	HP > NP, OP; OP > NP
Time in recovery	3.41 (1.92)	4.15 (1.92)	3.72 (1.75)	4.34*	0.02	HP > NP
Depression	9.03 (5.63)	5.42 (4.80)	9.42 (5.81)	19.53***	0.10	HP < NP, OP
Anxiety	8.44 (5.96)	4.99 (5.59)	8.17 (5.59)	12.79***	0.07	HP < NP, OP
Life satisfaction	15.71 (7.46)	22.04 (7.63)	20.27 (7.58)	19.79***	0.10	NP < HP, OP

*p < 0.05. **p < 0.01. ***p < 0.001. ANOVA, Analysis of Variance; NP, Non-passionate; HP, Harmoniously passionate; OP, Obsessively passionate. Games-Howell tests run *post hoc*.

### Regression analysis

To test the final research question, a hierarchical linear regression analysis first tested the hypothesis that passion type would predict RC and was found to be significant *F* (2,233) = 24.18, *p* < 0.001 accounting for 17% of the variance in recovery capital averages (see Table 5). The results revealed a significant relationship between HP and the ARC (*β* = 0.42, *p* < 0.001), however, the association between OP and the ARC was not significant (*β* = −0.07, *p* = 0.246). These findings are only partially aligned with our hypotheses, as we believed both HP and OP would have a significant relationship to RC. The data suggests that while passion type does play a role in the development of RC, HP has greater impact than OP. While we hypothesized that OP would undermine RC, the results indicate that OP is unlikely to contribute either positively or negatively to recovery resources.

**Table 5:** Regression analyses testing passion type on recovery capital with and without covariates.

Variable	*B*	95% CI for *B*	*SE B*	β	*R* ^2^	*∆R* ^2^
Step 1					0.17	
Constant	2.81***	[2.29, 3.32]	0.26			
HP	0.31***	[0.22, 0.40]	0.04	0.42***		
OP	−0.03	[−0.08, 0.02]	0.03	−0.07		
Step 2					0.63	0.46***
Constant	2.53***	[2.11, 2.94]	0.21			
HP	0.19***	[0.13, 0.26]	0.03	0.26***		
OP	−0.00	[−0.04, 0.04]	0.02	−0.00		
Time in Recovery	0.03*	[0.00, 0.07]	0.02	0.09*		
Depression	−0.01	[−0.03, 0.01]	0.01	−0.10		
Anxiety	−0.03***	[−0.05, −0.02]	0.01	−0.26***		
Life Satisfaction	0.05***	[0.04, 0.06]	0.00	0.50***		

*p < 0.05. **p < 0.01. ***p < 0.001.

The second step of the hierarchical model tested the final hypothesis that passion would continue to contribute to RC above and beyond other related variables and was significant *F* (6,229) = 64.69, *p* < 0.001 accounting for 63% of the variance (see Table 5). The results indicated a significant positive association between RC and HP (*β* = 0.26, *p* < 0.001), time in recovery (β = 0.09, *p* < 0.05), and satisfaction with life (*β* = 0.50, *p* < 0.001) along with a significant negative association between anxiety and RC (*β* = −0.26, *p* < 0.001). The final model confirmed that while passion plays a role in the development of RC, there are other variables that need to remain a consideration to best understand recovery outcomes.

## Discussion

Previous literature on the DMP has explored the effect of passion type, both harmonious and obsessive, on health behaviors including exercise and substance use, but has yet to apply it to addiction recovery Utilizing an online sample of adults in recovery, the current study aimed to test the association between passion type and recovery capital. We hypothesized that HP would be related to higher RC and contribute to RC over and above other related variables such as time in recovery, depression, anxiety, and satisfaction with life, expecting OP to reveal a converse relationship. Our hypotheses were partially supported by the data, finding that while there are significant differences in RC based on grouping individuals into HP engagement versus OP engagement, when estimating RC with the averages of HP and OP, only HP had a significant effect. This novel approach to understanding passion and recovery outcomes offers preliminary evidence for the relationship between HP and RC but leaves questions about how OP influences recovery.

Mean comparisons confirmed the association between passion and RC as individuals endorsing HP had significantly higher RC scores, lower depression, and reduced anxiety when compared to their peers endorsing OP and those without a passion. While these findings build upon previous research on the DMP and mental wellness, they add a novel approach to recovery literature. Harmonious engagement in a passion includes an autonomous internalization of the activity, high levels of persistence, self-growth, and an openness to experience the world in a mindful manner (10). The current study offers evidence that those qualities may be beneficial to individuals in recovery by amplifying positive outcomes (e.g., higher RC) compared to those who engage in OP which is based on rigid engagement in an enjoyable activity. The findings also highlight the differences between those who do not engage in a passion at all, as those individuals experienced significantly lower RC than both the HP and OP groups. This is evidence for the benefits of passionate engagement overall, while also recognizing that HP is related to the most adaptive outcomes. This finding is made evident by our regression results which continued to show that even when including time in recovery, mental health, and life satisfaction variables, HP is a significant predictor of RC.

Previous literature on passion has reported that the type of passion based on an uncontrollable urge to persistently engage in a passion (OP) has negative outcomes for individuals including conflict with other activities they enjoy, relationship distress, and negative affect (11). As such, we hypothesized that OP would negatively impact RC, however, our hypothesis was not supported by the data. It is possible, based on the result that those who did not engage in a passion at all had the lowest amount of RC, that engaging in a passion, even if it is obsessively, is still contributing to the resources an individual in recovery has. These resources include having an important activity in their life, feeling like part of a community, and feeling fulfilled in life, all components of passion that one may feel when they are engaged in the activity they enjoy. So, while research on OP has shown negative long-term effects on health and well-being, it is possible those were not captured in our cross-sectional analysis.

Previous research has explored concepts with similar components to passion such as the literature on adolescence which shows that time spent in pro-social activities (e.g., school clubs, performing arts) reduces subsequent risky substance use (27, 28) and increases positive development of pro-social group norms. Qualitative research on recovery also found that emerging adults in Norway believed meaningful activities in their communities promoted purpose and aided in building connections with others “introducing positive experiences and joy to the recovery process” (29). Unlike pro-social, substance-free activities, the DMP recognizes engagement in an activity that the individual has chosen to engage in persistently and has integrated into their personality, adding a level of motivation and commitment that is not found in the drug-free activities researched previously. Additionally, as reported by Hardy et al. (30) autonomous motivation, like that found in HP, is a key factor in pro-social engagement and health promotive behaviors.

Recovery research has explored how discovering meaning, and purpose can play a central role in the recovery process, similar to the outcomes found within the DMP literature. White (31) described the process of finding meaning and purpose as linking the past, present and future of one’s life which can be a form of recovery capital. In line with this research, studies have found that engagement in meaningful activities (e.g., work, volunteering) while in recovery contributes to recovery outcomes through the development of purpose, community engagement, and changes to one’s identity (32–34). Similar findings have been shown with the DMP, where passionate engagement, especially harmoniously, contributes to high levels of self-growth, social connection, and identity integration. The difference, however, is that research on meaningful activities does not always focus on activities that are motivating, self-defining, and of high value. While the current analyses did not account for the unique contribution of pro-social activities and purpose, future research may seek to test the similarities and differences found between them and passion to understand how passion accounts for distinct changes in recovery capital and other recovery outcomes.

The preliminary findings offer potential clinical implications including potential interventions aimed at assessing passion type and then leveraging passion to enhance recovery. Given that passions are present for 75–84% of the general population (11, 12), clinicians may be able to capitalize on the motivation, dedication, and identity integration already taking place in the client’s life. Clinicians could assess the presence of a passion in their client’s life when collaborating on clinical goals, and work to reignite an old passion or find a new passion by discussing an activity that the client loves to do and encouraging engagement. This passionate engagement can exist within their recovery to build personal skills, bring about positive affect, and connect with pro-social groups in the community. Using the DMP as another tool to build RC and increase wellness adds depth and variety to the field and may allow for many future research questions to be answered.

### Limitations and future research

While the findings of this study offer promise to further evaluation of passion and recovery, it is not without limitations. The participants of this study are limited, and homogeneous in race and ethnicity (82% Caucasian) which does not allow for generalizability of the findings to more diverse populations. Similarly, the sample was recruited through an online data crowdsourcing platform (MTURK), which provides the benefit of finding participants throughout the U.S. but is also limited to individuals with regular time and access to such methods. Online platforms such as Amazon’s MTURK have been criticized for the potential vulnerability to web robots (bots), socially desirable responses, and self-misrepresentation which we recognize is a limitation of the current study design. Nonetheless, research has shown that when proper steps are taken to ensure data integrity, as has been done in the current study, the risks are similar to other methods of data collection (35–37). Finally, we acknowledge that the study utilized a cross-sectional design and self-report measures, which limits our ability to draw causal inferences.

The field of passion and addiction recovery remains open to studies that implement various study designs, methods, and analysis. As such, future research should continue to explore the associations and potential implications of passion as a way of fostering recovery with larger and more diverse samples. Additionally, longitudinal study designs may be able to discern the specific effects of both HP and OP on recovery. The potential for clinical and policy implications also remains unclear, specifically what interventions can be used to assess and implement harmonious engagement for people in recovery. Therefore, future research should aim to examine these possibilities.

## Conclusion

This study presented a novel approach to addiction recovery research by examining the association between general passions, as defined by Vallerand’s DMP, and recovery outcomes. Our findings show that harmoniously passionate engagement has a positive relationship with recovery and satisfaction with life, while maintaining a negative relationship to negative mental health outcomes. These findings provide support for passion as a health promotive behavior that allows individuals to have an autonomous relationship with their health. As such, passion should remain a focus of future research examining the experience of addiction recovery.

## Data Availability

The raw data supporting the conclusions of this article will be made available by the authors, without undue reservation.

## References

[1] Substance Abuse and Mental Health Services Administration. Key substance use and mental health indicators in the United States: Results from the 2023 National Survey on drug use and health. (2024). Available at: https://www.samhsa.gov/data/report/2023-nsduh-annual-national-report (Accessed December 27, 2023).

[2] White WL. Slaying the dragon: The history of addiction treatment and recovery in America. 2nd ed Bloomington, IL: Chestnut Health Systems (2014).

[3] Kelly JF Hoeppner B. A biaxial formulation of the recovery construct. Addict Res Theory. (2015) 23:5–9. doi: 10.3109/16066359.2014.930132

[4] The Betty Ford Institute Consensus Panel. What is recovery? A working definition from the Betty ford institute. J Subst Abus Treat. (2007) 33:221–8. doi: 10.1016/j.jsat.2007.06.001, PMID: 17889294

[5] Witkiewitz K Wilson AD Pearson MR Montes KS Kirouac M Roos CR. Profiles of recovery from alcohol use disorder at three years following treatment: can the definition of recovery be extended to include high functioning heavy drinkers? Addiction. (2019) 114:69–80. doi: 10.1111/add.14403, PMID: 30063267 PMC6289769

[6] Substance Abuse and Mental Health Services Administration. Working Definition of Recovery: 10 Guiding Principles of Recovery. (2012). Available at: https://store.samhsa.gov/sites/default/files/d7/priv/pep12-recdef.pdf (Accessed October 8, 2024).

[7] Cloud W Granfield R. Natural recovery from substance dependency: lessons for treatment providers. J Soc Work Pract Addict. (2001) 1:83–104. doi: 10.1300/J160v01n01_07

[8] Cloud W Granfield R. Conceptualizing recovery capital: expansion of a theoretical construct. Subst Use Misuse. (2008) 43:1971–86. doi: 10.1080/10826080802289762, PMID: 19016174

[9] Vilsaint CL Kelly JF Bergman BG Groshkova T Best D White W. Development and validation of a brief assessment of recovery capital (BARC-10) for alcohol and drug use disorder. Drug Alcohol Depend. (2017) 177:71–6. doi: 10.1016/j.drugalcdep.2017.03.022 28578224

[10] Vallerand RJ Blanchard C Mageau GA Koestner R Ratelle C Léonard M. Les passions de l’âme: On obsessive and harmonious passion. J Pers Soc Psychol. (2003) 85:756–67. doi: 10.1037/0022-3514.85.4.756, PMID: 14561128

[11] Vallerand RJ. The dualistic model of passion In: The psychology of passion: A dualistic model. UK: Oxford University Press (2015)

[12] Philippe FL Vallerand RJ Lavigne GL. Passion does make a difference in People’s lives: a look at well-being in passionate and non-passionate individuals. Appl Psychol Health Well Being. (2009) 1:3–22. doi: 10.1111/j.1758-0854.2008.01003.x, PMID: 39910408

[13] Vallerand RJ. On passion for life activities. Adv Exp Soc Psychol. (2010) 42:97–193. doi: 10.1016/S0065-2601(10)42003-1

[14] Curran T Hill AP Appleton PR Vallerand RJ Standage M. The psychology of passion: a meta-analytical review of a decade of research on intrapersonal outcomes. Motiv Emot. (2015) 39:631–55. doi: 10.1007/s11031-015-9503-0

[15] Davis AK Arterberry BJ. Passion for marijuana use mediates the relations between refusal self-efficacy and marijuana use and associated consequences. J Psychoactive Drugs. (2019) 51:343–50. doi: 10.1080/02791072.2019.1596334, PMID: 30947640 PMC6764850

[16] Davis AK Arterberry BJ Bonar EE Bohnert KM Walton MA. Why do young people consume marijuana? Extending motivational theory via the dualistic model of passion. Translational Issues in Psycholog Sci. (2018) 4:54–64. doi: 10.1037/tps0000141, PMID: 29732383 PMC5931733

[17] Davis AK Rosenberg H. Application of the passionate attachment model to recreational use of MDMA/ecstasy. J Psychoactive Drugs. (2015) 47:24–9. doi: 10.1080/02791072.2014.973125, PMID: 25715069

[18] Dolan S Arterberry B Davis A. A quadripartite model of passion for marijuana use: associations with consumption, consequences, craving, and satisfaction with life. Addict Res Theory. (2021) 29:30–5. doi: 10.1080/16066359.2020.1718117, PMID: 33716599 PMC7954138

[19] Kelly JF Bergman B Hoeppner BB Vilsaint C White WL. Prevalence and pathways of recovery from drug and alcohol problems in the United States population: implications for practice, research, and policy. Drug Alcohol Depend. (2017) 181:162–9. doi: 10.1016/j.drugalcdep.2017.09.028, PMID: 29055821 PMC6076174

[20] Vallerand RJ Houlfort N. (eds). Passion at work: towards a new conceptualization In: Emerging perspectives on values in organizations. New York, NY: Oxford University Press (2003)

[21] Groshkova T Best D White W. The assessment of recovery capital: properties and psychometrics of a measure of addiction recovery strengths. Drug Alcohol Rev. (2013) 32:187–94. doi: 10.1111/j.1465-3362.2012.00489.x, PMID: 22882622

[22] Kroenke K Strine TW Spitzer RL Williams JBW Berry JT Mokdad AH. The PHQ-8 as a measure of current depression in the general population. J Affect Disord. (2009) 114:163–73. doi: 10.1016/j.jad.2008.06.026, PMID: 18752852

[23] Spitzer RL Kroenke K Williams JBW Löwe B. A brief measure for assessing generalized anxiety disorder: the GAD-7. Arch Intern Med. (2006) 166:1092–7. doi: 10.1001/archinte.166.10.1092, PMID: 16717171

[24] Diener E Emmons RA Larsen RJ Griffin S. The satisfaction with life scale. J Pers Assess. (1985) 49:71–5. doi: 10.1207/s15327752jpa4901_13, PMID: 16367493

[25] Lee S Lee DK. What is the proper way to apply the multiple comparison test? Korean J Anesthesiol. (2018) 71:353–60. doi: 10.4097/kja.d.18.00242, PMID: 30157585 PMC6193594

[26] Graham JW. Missing data analysis: making it work in the real world. Annu Rev Psychol. (2009) 60:549–76. doi: 10.1146/annurev.psych.58.110405.085530 18652544

[27] Eccles JS Barber BL. Student council, volunteering, basketball, or marching band: what kind of extracurricular involvement matters? J Adolesc Res. (1999) 14:10–43. doi: 10.1177/0743558499141003

[28] McCabe KO Modecki KL Barber BL. Participation in organized activities protects against adolescents’ risky substance use, even beyond development in conscientiousness. J Youth Adolesc. (2016) 45:2292–306. doi: 10.1007/s10964-016-0454-x, PMID: 26979446

[29] Bahl NKH Øversveen E Brodahl M Nafstad HE Blakar RM Ness O. In what ways do emerging adults with substance use problems experience their communities as influencing their personal recovery processes? J Community Psychol. (2022) 50:3070–100. doi: 10.1002/jcop.22816, PMID: 35187694 PMC9545888

[30] Hardy SA Dollahite DC Johnson N Christensen JB. Adolescent motivations to engage in pro-social behaviors and abstain from health-risk behaviors: a self-determination theory approach. J Pers. (2015) 83:479–90. doi: 10.1111/jopy.12123, PMID: 25130713

[31] White WL Laudet AB Becker JB. Life meaning and purpose in addiction recovery. Addiction Professional. (2006) 4:18–23.

[32] Best D Gow J Knox T Taylor A Groshkova T White W. Mapping the recovery stories of drinkers and drug users in Glasgow: quality of life and its associations with measures of recovery capital. Drug Alcohol Rev. (2012) 31:334–41. doi: 10.1111/j.1465-3362.2011.00321.x, PMID: 21615809

[33] Collinson B Best D. Promoting recovery from substance misuse through engagement with community assets: asset based community engagement. Substance Abuse: Res Treatment. (2019) 13:1178221819876575. doi: 10.1177/1178221819876575, PMID: 31598063 PMC6764067

[34] Veseth M Svendsen TS Nesvaag S Moltu C Davidson L Bjornestad J. And then the rest happened”—a qualitative exploration of the role that meaningful activities play in recovery processes for people with a diagnosis of substance use disorder. Subst Abus. (2022) 43:260–6. doi: 10.1080/08897077.2021.1941506, PMID: 34214010

[35] Aguinis H Villamor I Ramani RS. MTurk research: review and recommendations. J Manag. (2021) 47:823–37. doi: 10.1177/0149206320969787

[36] Hauser DJ Schwarz N. Attentive Turkers: MTurk participants perform better on online attention checks than do subject pool participants. Behav Res Methods. (2016) 48:400–7. doi: 10.3758/s13428-015-0578-z, PMID: 25761395

[37] Wessling KS Huber J Netzer O. MTurk character misrepresentation: assessment and solutions. J Consum Res. (2017) 44:211–30. doi: 10.1093/jcr/ucx053

[38] Thomas KA Clifford S. Validity and Mechanical Turk: An assessment of exclusion methods and interactive experiments. Comput Hum Behav. (2017) 77:184–97. doi: 10.1016/j.chb.2017.08.038

